# Quantifying the Impact of Capacity Constraints in Economic Evaluations: An Application in Precision Medicine

**DOI:** 10.1177/0272989X211053792

**Published:** 2021-10-25

**Authors:** Stuart J. Wright, William G. Newman, Katherine Payne

**Affiliations:** Manchester Centre for Health Economics, The University of Manchester, Manchester, Greater Manchester, UK; Manchester Centre for Genomic Medicine, Manchester University NHS Foundation Trust, Manchester, Greater Manchester, UK; Evolution and Genomic Sciences, School of Biological Sciences, University of Manchester, Manchester, Greater Manchester, UK; Manchester Centre for Health Economics, The University of Manchester, Manchester, Greater Manchester, UK

## Abstract

**Background:**

Examples of precision medicine are complex interventions featuring both testing and treatment components. Because of this complexity, there are often barriers to the introduction of such interventions. Few economic evaluations attempt to determine the impact of these barriers on the cost-effectiveness of the intervention. This study presents a case study economic evaluation that illustrates how the value of implementation methods may be used to quantify the impact of capacity constraints in a decision-analytic model.

**Methods:**

A baseline decision-analytic model-based economic evaluation of *ALK* mutation testing was reproduced from a published technology appraisal. Three constraints (commissioning awareness, localization of testing, and pathology laboratory capacity) were identified using qualitative interviews, parameterized, and incorporated into the model. Value of implementation methods were used alongside incremental cost-effectiveness ratios (ICERs) to quantify the impact on the cost-effectiveness and net monetary benefit (NMB) of each capacity constraint and from the 3 constraints combined.

**Results:**

Each of the 3 capacity constraints resulted in a loss of NMB ranging from £7773 (0.1% of the total) per year for localized testing to £4,907,893 (77%) for a lack of awareness about commissioning *ALK* testing. When combined, the constraints resulted in a loss of NMB of £5,289,414 (83%). The localization and limited pathology capacity constraints slightly increased the ICER, but the lack of commissioning awareness constraint did not change the ICER.

**Conclusions:**

Capacity constraints may have a significant impact on the NMB produced by examples of precision medicine. Value of implementation methods can be used to quantify the impact of such constraints by combining the impact of the constraints on the cost-effectiveness of the intervention with the impact on the number of patients receiving the intervention.

**Highlights:**

- While capacity constraints may prevent the use of precision medicine in clinical practice, economic evaluations rarely account for the impact of such barriers.
- This study demonstrates how constraints can be identified using qualitative methods and subsequently incorporated into decision-analytic models using quantitative value of implementation methods.
- In addition, this article demonstrates how value of implementation methods can be used to account for the impact of capacity constraints on the costs and benefits of an intervention as well as the number of patients receiving the intervention.
- In the case study presented herein, a capacity constraint reducing patient access to an example of precision medicine caused the biggest loss of net monetary benefit.
- Health economists should consider moving beyond incremental cost-effectiveness ratios to measures of total net monetary benefit to fully capture the impact of implementing precision medicine.

Driven by the need to understand the opportunity cost of introducing new health care interventions, or adapting existing ones, methods of economic evaluation, in general, and model-based cost-effectiveness analysis (CEA) have become standard approaches to generate evidence for decision makers.^1^ Methods of economic evaluation take account of one constraint in the health care system: the budget available to spend. Current applications of CEA do not identify or quantify the impact of multiple capacity constraints in the health system to deliver a new intervention.^2,3^ The term *capacity constraint*, beyond the constraint of dealing with a set health care budget, is not a well-defined concept. Here, we define capacity constraints as “any factor which impedes or limits the amount of health status produced for a population of patients receiving specified interventions, or policies, provided by the healthcare system.”^4^ Examples of health system capacity constraints may include a lack of resources such as laboratory testing machinery or sufficiently trained medical staff but could also include more abstract barriers to interventions such as out-of-date clinical guidelines. Brennan et al.^3^ suggested the importance of capturing the impact of different health care system capacity constraints when describing a taxonomy of decision-analytic models: “Inaccurate cost-benefit assessments can result from ignoring the interactions between service capacity decisions, waits and health outcomes.”

Precision medicine is an emerging health care intervention attracting much attention by funding bodies and decision makers.^5–7^ Precision medicine is underpinned by the promise of the more effective use of health care budgets by directing interventions to those most likely to accrue positive health benefits.^8^ Capacity constraints in the health care system may impede the provision of a new precision medicine to all potentially eligible patients. For example, when gefitinib was approved by the National Institute for Health and Care Excellence (NICE) in 2010 for patients with *EGFR* mutation-positive non–small-cell lung cancer, there was limited testing capacity in England.^9^ For a number of years following the approval of the medicine, patients had limited access to EGFR testing and therefore could not receive a medicine that could have improved their quality and length of life.^10^ Taking an economic perspective, this means that if some patients do not receive a potentially cost-effective intervention, then the maximum total incremental societal health gain from introducing the intervention may not be achieved.^11^

Capacity constraints may also have a significant impact on cost-effectiveness if they affect the cost or consequences produced by the intervention. This situation may occur if the constraint increases the cost of providing the intervention to patients or reduces the expected benefit for each patient.^12^ For example, if clinicians need to learn new techniques to effectively use an intervention, but a capacity constraint means that they see few patients each year, the benefits per patient and therefore the cost-effectiveness of the intervention may be reduced. In such cases, capacity constraints will affect the size of the change in societal health but also mean that there could be a net reduction in the total health produced by the system instead of a net gain. Within this context, this study aimed to describe and apply an approach to including multiple health care system capacity constraints that builds on existing methods of decision-analytic model-based CEA.

## Methods

This study conceptualized and built a decision-analytic model in line with published recommendations^13^ and reporting criteria.^14^ This study used an exemplar case study to illustrate the process of incorporating and quantifying the impact of capacity constraints in a decision-analytic model-based CEA. The decision-analytic model was built to allow the inclusion of specific capacity constraints relevant to the defined decision problem (see Table 1).

**Table 1:** Key Design Criteria

Decision problems	What is the cost-effectiveness and net monetary benefit of *ALK* testing to guide treatment with crizotinib?
Population	Patients with stage III or IV, *EGFR*-negative non–small-cell lung cancer
Intervention	Anaplastic lymphoma kinase (*ALK*) testing that uses IHC and fluorescence in situ hybridization to target treatment with an appropriate treatment such as an *ALK* inhibitor (crizotinib)
Comparator	No testing and a chemotherapy agent (docetaxel)
Model type	Linked decision tree (testing component) and Markov model (treatment component)
Setting and perspective	Hospital setting; NHS England
Time horizon	Lifetime for this population; 15 y
Costs	National currency (£) at 2014 prices
Consequences	Quality-adjusted life-years (QALYs)
Discounting	3.5% for both costs and consequences
Decision rule	Incremental cost per QALY should fall under £50,000 to be deemed a cost-effective use of resources. This decision rule is consistent with end-of-life criteria invoked by NICE^a^ for this decision problem as the intervention meets: List 3 criteria: • The treatment is for patients who are expected to live for less than 24 mo • The treatment is expected to offer at least 3 additional months of life • The treatment is licensed for a small patient population

ICER, incremental cost-effectiveness ratio; IHC, immunohistochemistry; NHS, National Health Service; NICE, National Institute for Health and Care Excellence; QALYs, quality-adjusted life-years.

a NICE end-of-life criteria.^15^

### Case Study

The selected case study for this work was a NICE Single Technology Appraisal (TA) and published as part of NICE TA296.^16^ The model in this TA sought to evaluate the cost-effectiveness of crizotinib for patients with *ALK* mutation–positive non–small-cell lung cancer compared with standard chemotherapy (docetaxel). The use of an existing model allows the comparison of the cost-effectiveness of the intervention in the presence of capacity constraints with the estimated cost-effectiveness of the intervention in the absence of constraint. An example was chosen from the field of precision medicine, as such interventions are by their nature complex interventions and face many barriers to their use in clinical practice.^4,17^

### Incorporating and Quantifying the Impact of Capacity Constraints

The process for incorporating and quantifying the impact of capacity constraints developed in this study has 5 key steps:

1. Conceptualize and build a decision-analytic model to evaluate the cost-effectiveness and net monetary benefit of the intervention in the absence of capacity constraints.
2. Identify specific capacity constraints relevant to the introduction of the intervention under evaluation
3. Incorporate the selected capacity constraints into the decision-analytic model
4. Parameterize the selected capacity constraints in the decision-analytic model
5. Quantify the impact of the constraints on the relative cost-effectiveness of the intervention under evaluation

### Decision-Analytic Model

**Figure 1:**
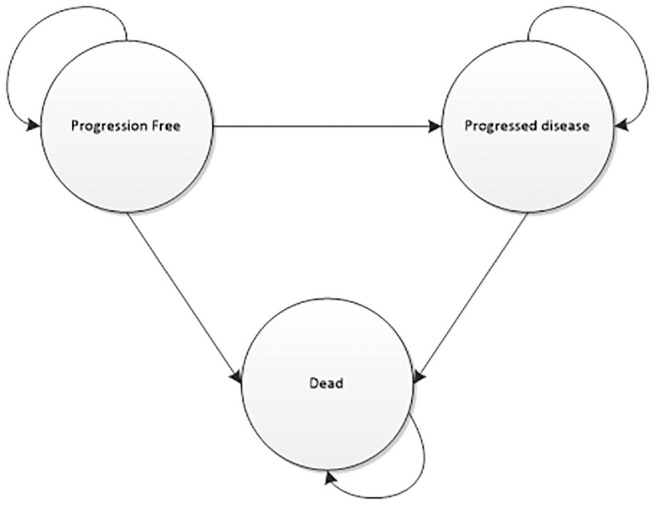
Markov model.

The decision-analytic model was conceptualized and built to replicate a published model (see Supplementary Appendix 1). The original model was submitted by the manufacturer of crizotinib (Pfizer) to NICE as part of the process of NICE TA296. In this case study, the original submitted model, before any requested changes by the Evidence Review Group, was used as the baseline analysis. A fully executable version of this model is not in the public domain, but a description of the model was identified in the published documents that supported the NICE TA.^18^ As part of this study, it was therefore necessary to conceptualize and rebuild the decision-analytic model de novo from this description (Figure 1).

**Figure 2:**
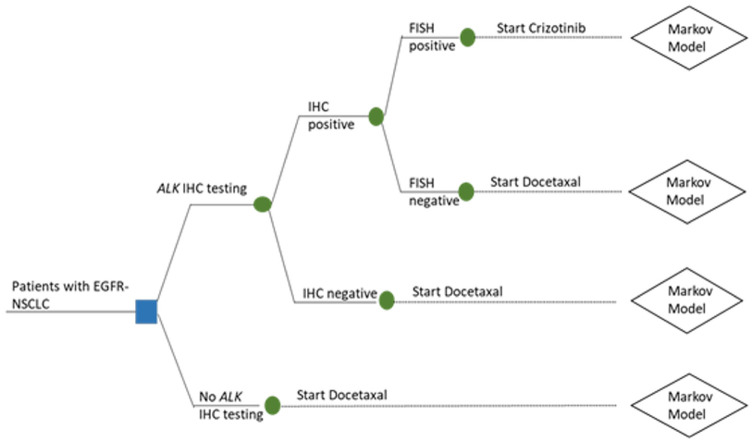
Decision tree.

The manufacturer submission focused on evaluating the cost-effectiveness of crizotinib as opposed to *ALK* testing and therefore contained only a 3-state Markov model representing the experiences of patients receiving crizotinib or docetaxal. A bespoke decision tree was appended to the Markov model to represent the *ALK* testing pathway, as this was not included in the original manufacturer’s submission (Figure 2). As only minimal details of the testing component were provided in the documentation supporting NICE TA296, additional information was taken from relevant published economic evaluations identified in a meta-review of economic evaluations of precision medicine.^4,19–21^

### Identifying Relevant Capacity Constraints

The relevant capacity constraints were defined as barriers to implementing *ALK* mutation testing. These barriers were identified in semistructured telephone interviews that aimed to identify the barriers that had been faced in introducing existing examples of precision medicine for non–small-cell lung cancer (NSCLC).^22,23^ In brief, this process involved interviewing 10 clinical experts (5 medical oncologists, 3 pathologists, 2 geneticists, and 1 service commissioner) with experience of implementing such interventions. A modified framework analysis was used to analyze the data, resulting in the identification of 17 barriers grouped into 5 themes: the managed entry of precision medicine for NSCLC, the commissioning and reimbursement of precision medicine for NSCLC and specifically the test component of precision medicine, the complexity of the logistics around providing tests, opinions about whether test provision should be localized or centralized, and opinions about future developments, including potential barriers to their introduction, in precision medicine for NSCLC.

These 5 themes were used as the starting point to select specific capacity constraints relevant to the stated decision problem (Table 1). After reviewing the qualitative data, 3 key exemplar capacity constraints were selected by the research team using 2 criteria: potential impact on the implementation of the intervention and its cost-effectiveness and ability of the NHS to potentially address the constraints through investment.

1. Lack of awareness about how *ALK* testing was commissioned. Clinical experts interviewed suggested that there was a lack of understanding about how *ALK* testing was supposed to be reimbursed. This lack of clarity resulted in some instances in which no testing was conducted and some instances in which testing was conducted but a financial loss may have been experienced by the hospital trust providing the test. These 2 potential actions may result in different potential economic impacts. If laboratories did not offer testing so as not to experience a financial loss, then a number of patients who may have benefitted from targeted treatment may have received the less effective chemotherapy as they could not receive testing. If hospital trusts offered the testing at a financial loss, then patients would have received testing but other patients receiving interventions from the same budget may have experienced a health loss from a lack of available funding for other existing interventions. In this study, the former approach, whereby it is assumed that patients are not provided with testing due to the constraint, was assumed to take place. This decision was made to demonstrate how the methods proposed in this study can be used to evaluate the impact of constraints that affect the number of patients receiving an intervention as well as the impact of constraints that affect the costs or benefits of the intervention.
2. Degree of centralization of immunohistochemistry (IHC) testing. When the intervention was initially introduced, some *ALK* testing was performed in larger centralized laboratories, whereas some was performed in smaller, local laboratories. Because of the volume of requests for *ALK* testing received at centralized laboratories, turnaround times were potentially slow. It was believed by some clinical experts that localizing IHC testing to the hospitals where most patients are based could reduce turnaround times, ensuring there is a shorter delay in patients starting treatment. Others believed that centralized testing resulted in economies of scale, reducing the cost per patient. Some believed that centralized testing meant that pathologists gained more experience in conducting tests and that this might lead to a better quality of testing than if testing were localized. It is not clear what the impact of using localized or centralized testing would have on the relative cost-effectiveness of *ALK* testing for crizotinib.
3. Ability to conduct *ALK* testing dependent on whether centralized or localized services were used. The clinical experts suggested that a lack of financial and human resources meant that it was difficult to offer responsive, high-quality testing. The actual impact of limited pathology laboratory capacity is difficult to enumerate. One assumption is that the negative impacts of either centralizing or localizing pathology testing occur as a result of the limited ability, because of financial and human resource constraints, of pathology laboratories to deliver responsive and high-quality testing. Specifically, the longer turnaround times faced by centralized laboratories and the more expensive, lower-quality tests associated with localized testing are a product of the capacity of these laboratories.

Intuitively, it can be seen that constraints 2 and 3 are closely interrelated. Constraint 2 can be interpreted as the lost net benefit resulting from the current mixture of localized and centralized testing given the current restricted level of pathology laboratory capacity. This net benefit can also be characterized as the value of fully localizing or centralizing testing while pathology laboratory capacity remains constrained. Constraint 3 can be interpreted as the lost net benefit due to limited pathology laboratory capacity given the current mixture of localized or centralized testing. This constraint can also be characterized as the potential value of investing in improving pathology lab capacity while the mixture of localized and centralized testing remains fixed.

### Incorporating Capacity Constraints in the Decision-Analytic Model

**Figure 3.**
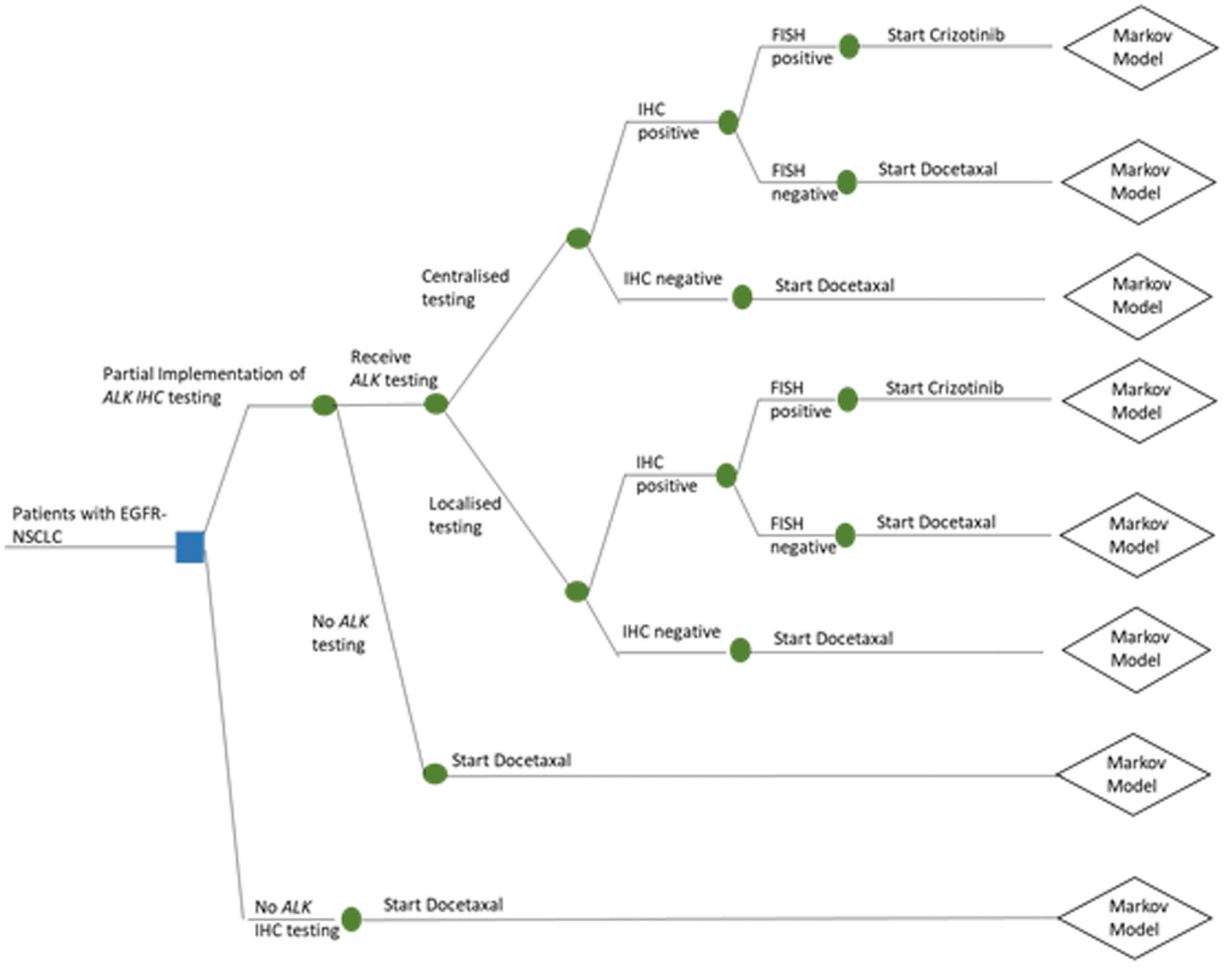
Decision tree incorporating capacity constraints.

To incorporate the 3 selected capacity constraints into the decision-analytic model-based CEA of *ALK* testing and targeted treatment, the structure of decision tree was adapted (see Figure 3). New decision problems were outlined for the 3 capacity constraints separately and combined (see Table 2). Full technical validation of the base-case model was beyond the scope of this study.^24^ However, to check the validity of the restructured model, all constraints were set to zero. In this situation, the model was expected to represent the original baseline model and produce identical results to those in NICE TA296. The decision-analytic model structure allows the number of patients to access commissioned IHC to be represented. If 100% of patients have access to IHC, then the decision problem becomes the same as that specified in NICE TA296. If there is no limit to pathology capacity and testing is centralized, then the model reverts to the structure to address decision problem 1. Testing must be centralized as opposed to localized for the model to revert into the base-case model, as it is assumed that localized IHC testing is more expensive.

**Table 2:** Decision Problems for the Decision-Analytic Model Incorporating Capacity Constraints

Decision problems	Decision problem 1: What is the cost-effectiveness and net monetary benefit of *ALK* testing to guide treatment with crizotinib given that some pathology laboratories are unaware of how *ALK* testing is commissioned? Decision problem 2: What is the cost-effectiveness and net monetary benefit of *ALK* testing to guide treatment with crizotinib given that some *ALK* IHC testing is conducted in centralized pathology laboratories and some is conducted in localized pathology laboratories? Decision problem 3: What is the cost-effectiveness and net monetary benefit of *ALK* testing to guide treatment with crizotinib given that some *ALK* IHC testing is conducted in centralized pathology laboratories and some is conducted in localized pathology laboratories and that all types of pathology laboratories are under-resourced? Decision problem 4: What is the cost-effectiveness and net monetary benefit of *ALK* testing to guide treatment with crizotinib given that some pathology laboratories are unaware of how *ALK* testing is commissioned, some *ALK* IHC testing is conducted in centralized pathology laboratories, and some is conducted in localized pathology laboratories and that all types of pathology laboratories are under-resourced?

IHC, immunohistochemistry.

### Parameterizing the Capacity Constraints

The inclusion of capacity constraints meant that additional parameter values needed to be identified and incorporated. Each constraint had the potential to influence the probability of patients experiencing different events or health states in the model, the costs of testing and treating patients, and the outcomes they experienced. To identify potential values for these new parameter values, pragmatic, targeted searches were conducted in the Embase and MEDLINE databases (in 2019) using the Ovid search tool.^25^ Key terms included in the search included “ALK,”“anaplastic lymphoma kinase,”“provision,”“availability,”“access,”“capacity,”“barrier,” and “patholog*.” Gray literature reports such as the National Lung Cancer Audit and Cancer Research UK reports, were also used to identify potential parameter values (Table 3).^10,26–28^

**Table 3:** Parameter Values for the Identified Capacity Constraints

Parameter	Constraint	Base-Case Value	Distribution	Sources	Assumptions
Probabilities					
Proportion of patients at trusts where there is an awareness of test commissioning arrangements	1) Lack of awareness about how *ALK* testing was commissioned	0.23	Beta∼(498,1660)	Ess et al. (2017)^29^ and Lee et al. (2018)^30^	Synthesis of *ALK* testing rates between 2008 and 2013 for 9 countries
Poportion of patients at trusts with access to localized testing	2) Degree of centralization of IHC testing 3) Ability to conduct *ALK* testing dependent on whether centralized or localized services were used	0.11	Beta∼(19,158)	National Lung Cancer Audit 2014^31^ and NHS Improvement 2019^32^	The 2014 National Lung Cancer Audit suggested that 27%^33^ of trusts offered in-house testing for *ALK*. However, this figure cannot be used as the parameter because under a centralized test provision, there will be a correct number of central pathology labs that offer the test, which will correspond to a localization rate of 0%. A recent strategy by NHS improvement seeks to consolidate the UK pathology laboratories into 29 networks. It is assumed in this study that each network will have 1 laboratory that offers *ALK* testing. Therefore, in this study, it was estimated that 19 trusts offered testing that was not specialized in *ALK* testing. If patients are distributed evenly across trusts, then this means 11% would receive localized testing.
Hazard ratio of crizotinib PFS (local) to crizotinib PFS (central)	2) Degree of centralization of IHC testing 3) Ability to conduct *ALK* testing dependent on whether centralized or localized services were used	0.822	Uniform∼(0.697,1)	Blackhall et al. 2017^34^	
Hazard ratio of crizotinib OS (local) to crizotinib OS (central)	2) Degree of centralization of IHC testing 3) Ability to conduct *ALK* testing dependent on whether centralized or localized services were used	0.775	Uniform∼(0.633,1)	Blackhall et al. 2017^34^	
Utilities					
Quality-of-life loss due to anxiety from delaying treatment due to test delay	2) Degree of centralization of IHC testing 3) Ability to conduct *ALK* testing dependent on whether centralized or localized services were used	0.03	Triangular∼(0, 0.1, 0.03) Original study does not present information from which distribution could be inferred	Moseholm et al. (2016)^35^	Original value from health-related quality-of-life gain after confirmed cancer diagnosis; assumed similar anxiety is experience while waiting for treatment start
Costs					
Cost of an extra appointment with an oncologist due to test delay	2) Degree of centralization of IHC testing 3) Ability to conduct *ALK* testing dependent on whether centralized or localized services were used	£101	Fixed	PSSRU Unit Costs of Health and Social Care (2016)^36^	
Cost of IHC testing when conducted in a local lab	2) Degree of centralization of IHC testing 3) Ability to conduct *ALK* testing dependent on whether centralized or localized services were used	£29	25 × (1 + uniform∼[0.1,0.25]) The original study does not present information from which value could be derived	Buckell et al. (2015)^37^	Cost is 17% higher than that in centralized labs due to inefficiency

IHC, immunohistochemistry; NHS, National Health Service; OS, overall survival; PFS, progression-free survival.

#### Awareness of ALK testing commissioning

To parameterize the “commissioning awareness” constraint, an estimate of how many NHS trusts were aware of how to commission testing and who therefore provided *ALK* testing was required. In the absence of available data on the degree of knowledge of commissioning awareness, the proportion of eligible patients receiving *ALK* testing in 2014 was used as a proxy.

A pragmatic literature search identified a single relevant article that had examined the availability of *ALK* testing in 9 countries between 2011 and 2013.^30^ For this case study, a weighted average of these uptake estimates (23%) was taken to provide an estimate of the availability of *ALK* testing in the United Kingdom. It was assumed that the commissioning awareness capacity constraint had no other effects on the model probabilities, costs, or quality-adjusted life-years (QALYs).

#### Centralization of IHC testing

The probability of patients receiving testing at a hospital that offered testing in house (localized testing) or through another laboratory (centralized testing) was informed by the National Lung Cancer Audit. In the National Lung Cancer Audit conducted in 2014, it was reported that 27% of trusts offered *ALK* testing in house.^31^ As there were 176 trusts at the time of the report, this suggests that 48 trusts had in-house *ALK* testing in 2014. Under a policy of centralization, there will be a reduced, optimum, number of pathology labs offering testing. Given the recent move to consolidate pathology laboratories into 29 networks in the United Kingdom, it was assumed that one laboratory in each network would offer *ALK* testing in an ideal scenario.^32^ Therefore, it is assumed that under full centralization, *ALK* testing would be offered by 29 laboratories and by 176 laboratories under full localization testing. In this example, it is assumed that patients are distributed evenly between laboratories, meaning that 11% (19 of 176) of patients would receive localized testing and 89% would receive centralized testing in the base case.

In the qualitative interview study, some participants believed that testing would be cheaper if it was performed in centralized laboratories. One identified study of the cost of pathology laboratories suggested that centralizing testing could reduce pathology costs by up to 17%.^37^ To reflect this, the cost of IHC testing for patients receiving localized testing was inflated by 17% to a value of £29.25.

The potential negative aspect of localized testing was identified as potentially poorer quality *ALK* testing. The potential impact on survival of reduced test quality in localized laboratories was modeled using data from the PROFILE 1005 study, which was included as a key source of evidence in the NICE TA of crizotinib.^16,18,34^ In the trial providing evidence of clinical effectiveness of crizotinib, *ALK* testing to determine patient eligibility was originally conducted in central laboratories before later being rolled out to localized laboratories. Patients in the local *ALK* testing group had lower median progression-free survival (PFS; 6.9 v. 8.4 months) and overall survival (OS; 16.9 v. 21.8 months) than those in the centralized group. It is assumed in this study that the differences in PFS and OS between patients whose tests were processed in localized or centralized laboratories is solely caused by the quality of the testing process in these laboratories.

To incorporate these effects, the transition probabilities for the state transition Markov model associated with patients receiving localized testing were altered. Simple hazard ratios were calculated for PFS (0.822) and OS (0.775) based on the ratio of the median survival in months. These estimates were applied to the transition probabilities between the progression-free, progressive disease, and dead health states used in the state transition Markov model.

While centralized laboratories were perceived to provide better quality tests, concerns were raised about their ability to provide a rapid turnaround time for test results. This delay may mean patients have to start their cancer treatment later as they are waiting for test results. A cost of £101 was assigned to patients receiving centralized *ALK* testing based on the cost of an assumed additional hour-long consultant oncologist appointment to discuss the test results and determine the appropriate treatment.^38^

A rapid review identified 1 relevant article that suggested that patients’ health-related quality of life (HRQoL) increases by 0.03 on receiving a definitive cancer diagnosis.^35^ It was assumed that the impact of delaying the start of treatment would have the same-size impact as waiting for a cancer diagnosis. A disutility of 0.03 over 9 days was therefore applied to the HRQoL for patients receiving centralized testing to represent the anxiety of having to delay their treatment start.

#### Use of centralized or localized testing services

It was assumed that the negative effects of centralized or localized *ALK* testing occurred because of the limited capacity of the pathology laboratories. If there was sufficient capacity in centralized laboratories, then tests could be turned around quickly enough to mean that patients would not experience anxiety from delayed treatment start and would not have an additional visit with a consultant. If testing was localized, then fully resourced pathology laboratories would be able to conduct testing of equal quality to that of centralized laboratories.

### Quantifying the Impact of Capacity Constraints

The base-case analysis incorporating capacity constraints took part in 2 stages. The first stage of the base-case analysis determined the impact of each of the 3 individual capacity constraints without the others present. The second stage of the base-case analysis determined the combined impact of all 3 capacity constraints simultaneously. Table 4 describes the value taken by each variable when determining the impact of each constraint individually or combined. In each of the 2 stages of analysis, the incremental expected costs and expected QALYs for the intervention were calculated. These incremental costs and QALYs were used to generate the incremental cost-effectiveness ratio (ICER) for the intervention. The ICER was estimated for the model in the presence of each capacity constraint individually and for all 3 combined.

**Table 4:** Value for Each Capacity Constraint in Each Scenario

Capacity Constraint	Parameter Values^a^		
	α	β	γ
Lack of commissioning awareness	0.23	1 or 0	1
Localization or centralization	1	0.11	1
Insufficient pathology staffing	1	1 or 0	0
All constraints combined	0.23	0.11	0

a Variable α is used to represent the lack of commissioning awareness constraint set, with a value of 1 meaning all patients are treated in hospitals who are aware of testing and a value of 0 meaning that no patients are treated in hospitals who are aware of testing. Variable β represents the localization or centralization constraint set, with a value of 1 representing a situation in which all testing is offered through localized testing, while a value of 0 represents a situation in which all testing is provided by centralized laboratories. It is not known whether localization of centralization is the capacity constraint; equation 1 contains 2 potential values for β in the calculation of the net monetary benefit in the absence of capacity constraints. This means that the value of β (1 or 0) that maximizes this value will need to be found, with the other value representing the capacity constraint. Variable γ represents the staffing level of pathology laboratories set at a value of 1, which represents a situation in which pathology laboratories are fully staffed; a value of 0 represents the level of pathology laboratory staffing in 2014.

The static value of implementation method was used to quantify the impact of the 3 capacity constraints.^39^ This method uses the total net benefit as the primary output of the analysis and therefore incorporates not only the cost-effectiveness of the intervention but also the number of patients treated. As the capacity constraints outlined in this study may affect the incremental marginal cost and benefits of the intervention, the method proposed by Wright et al.^40^ was used to allow for varying net monetary benefit. The impact of a capacity constraint can be quantified by subtracting the total net benefit provided by introducing the intervention when the constraint, or all of the constraints, are in place from the total net benefit provided when no constraints are in place. This value is the value of perfect implementation and is calculated using equation 1:

(1) $$\begin{array}{c} \mathrm{Value}\;\mathrm{of}\;\mathrm{Perfect}\;\mathrm{Implementaiton}=\\ n\left(\mathrm{NM}B_{\alpha =1,\beta =1\;\mathrm{or}\;0,\gamma =1)}-p_{\alpha ,\beta ,\gamma }.\mathrm{NM}B_{\alpha ,\beta ,\gamma }\right) \end{array}$$

where *n* is the total population of patients who can be tested, NMB_α=1,β=1 or 0,γ=0_ is the net monetary benefit per patient in the absence of the constraints, p_α,β,γ _is the proportion of patients receiving the intervention in the presence of the capacity constraints, and NMB_α,β,γ_ is the net monetary benefit of the intervention in the presence of the constraints. The variable α is used to represent the lack of commissioning awareness constraint. The variable β represents the localization or centralization constraint. The variable γ represents the staffing level of pathology laboratories.

### Sensitivity Analysis

Parameter uncertainty in the estimated size and effect on costs and QALYs of the capacity constraints was incorporated using probabilistic sensitivity analysis.^41,42^ Distributions were applied to the parameters and the analysis run 1000 times with new values drawn from the distributions in each iteration. Full details of the distributions used in the sensitivity analysis and any underlying assumptions are available in Table 3. The sample of incremental costs and QALYs estimated from the capacity constraint model probabilistic sensitivity analysis were plotted on an incremental cost-effectiveness plane. On the same figure, the distribution of incremental costs and QALYs resulting from the baseline model was also plotted. The proportion of iterations with positive NMB was calculated to estimate the probability that the intervention would be cost-effective in the presence and absence of capacity constraints.

## Results

In the base-case analysis, the use of *ALK* testing to guide treatment with crizotinib or docetaxel had an incremental cost of £1391 (using a price year of 2014) and provided an additional 0.035 QALYs per patient tested. This yielded an ICER for the intervention of £39,198 per QALY, which is similar to that produced by the source model (£41,554 per QALY). More information about the results and validity of the baseline model can be found in Supplementary Appendix 1. Given this ICER, there is evidence to suggest that *ALK* testing and treatment with crizotininb or docetaxel is cost-effective assuming the NICE end-of-life threshold of £50,000 per QALY. The probabilistic sensitivity analysis suggested that there was a 86% chance that the intervention was cost-effective at this threshold. At this threshold level of cost-effectiveness, the intervention would provide a net monetary benefit of £6,373,887 per year in the United Kingdom.

### Impact of Individual Capacity Constraints

To determine the face validity of the model, all of the capacity constraints were turned off in the restructured model to ensure that the same results were generated as in the baseline model. Setting the commissioning and pathology constraints to zero and offering fully centralized IHC testing yielded the same ICER and NMB figures as the baseline model, indicating that no errors in the underlying evaluation had been created by restructuring the decision model.

Table 5 summarizes the ICERs, NMB (current value of implementation), and NMB lost (value of perfect implementation) due to the presence of each constraint individually and together. In the first stage of the analysis, the ICER and impact on NMB of each capacity constraint individually was evaluated. The lack of commissioning awareness constraint did not affect the ICER of the intervention but resulted in a loss of NMB of £4,907,893. The localization constraint resulted in an intervention with a slightly higher ICER of £39,211 but a loss of NMB of only £7773. Finally, the pathology laboratory staffing constraint raised the ICER of the intervention to £40,322 and reduced the NMB by £808,746.

**Table 5:** Impact of the Inclusion of Capacity Constraints on Incremental Cost-Effectiveness Ratios and Net Monetary Benefit

Capacity Constraints	Value of Constraint	ICER^a^	Annual NMB^b^	Annual Societal QALY Gain	Annual NMB Shortfall due to Constraint^c^	Annual QALY Shortfall due to Constraint^d^
None (baseline)	100% of trusts aware of commissioning arrangements 0% of testing is localized/100% is centralized No impact of limited capacity on costs or outcomes	£39,198 per QALY gained	£6,373,887	124.48	£0 (0%)	0 (0&)
Lack of awareness of *ALK* commissioning	23% of trusts aware how to commission tests	£39,198 per QALY gained	£1,465,994	29.32	£4,907,893 (77%)	98.16 (77%)
Level of localization of test	11% of testing done in house	£39,211 per QALY gained	£6,366,114	127.32	£7,773 (0.1%)	0.16 (0.1%)
Level of pathology lab capacity	Impact of capacity on costs and outcomes of testing	£40,322 per QALY gained	£5,565,141	111.30	£808,746 (13%)	16.17 (13%)
Expected level of all constraints in 2014	23% of trusts aware how to commission tests 27% of tests done in house There is an impact of limited path capacity on costs and outcomes	£41,413 per QALY gained	£1,084,473	21.69	£5,289,414^e^ (83%)	105.79^1^ (83%)

ICER, incremental cost-effectiveness ratio; NMB, net monetary benefit; QALY, quality-adjusted life-year.

a ICER for *ALK* testing to guide crizotinib treatment with the health system capacity constraint in place compared with no testing and universal docetaxel.

b Net monetary benefit.

c NMB shortfall = NMB without constraints – NMB with 1 or all constraints present.

d QALY shortfall = QALYs without constraints – QALYs with 1 or all constraints present.

e The total NMB and QALY loss from the presence of all constraints is not a sum of that from individual constraints due to the interaction between localization and pathology laboratory capacity.

To determine whether localization or centralization of testing was the capacity constraint, the results produced when testing was fully localized or centralized were compared when no other constraints were present. Full localization of IHC testing yielded a marginally higher ICER (£39,317 per QALY) and reduced net benefit (£6,303,222 per year) due to the small additional cost of IHC testing in local laboratories. A comparison of the cost-effectiveness of the intervention under full localization or centralization in the presence of the other constraints also suggested that localized testing was the capacity constraint and that centralized testing yielded greater benefits for society.

### Impact of Capacity Constraints Combined

When combined, the 3 constraints resulted in an ICER of £41,413 and a loss of NMB of £5,289,414. This represents a loss of 83% of the potential benefit of the intervention.

### Sensitivity Analysis

**Figure 4:**
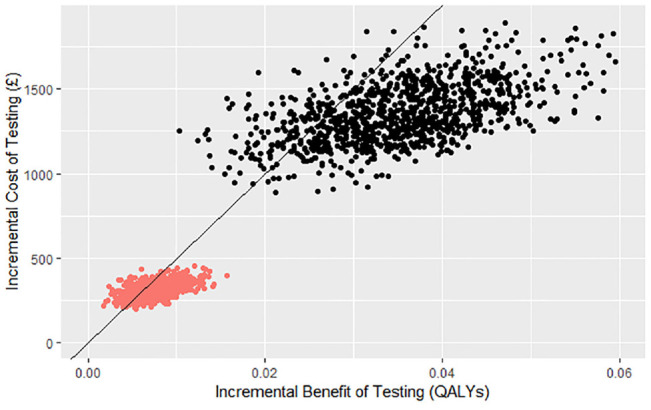
Incremental cost-effectiveness plane.

The incremental cost-effectiveness plane (see Figure 4) features the plotted incremental costs and benefits calculated from 1000 Monte Carlo simulations of the cost-effectiveness of *ALK* testing and crizotinib in the presence and absence of all 3 selected capacity constraints. The expected incremental costs and QALYs in the decision-analytic model including capacity constraints are lower as only a relatively small proportion of patients receive testing, and subsequent crizotinib, due to the lack of commissioning awareness in pathology laboratories. The effect of the localization and pathology capacity constraints is to reduce the probability of cost-effectiveness from 86% to 78%, showing there is an increase in the observed uncertainty in the model.^43^

## Discussion

This study demonstrates how current approaches to decision-analytic model–based economic evaluation of examples of precision medicine may overestimate their potential benefit by not accounting for capacity constraints. To guide the informed implementation of examples of precision medicine, it is necessary to quantify the potential impact of such constraints on the cost-effectiveness and net benefit of the intervention.

This study has shown how a mixed-methods approach incorporating both qualitative and quantitative research can be used to identify, incorporate, and quantify the impact of capacity constraints. There is emerging interest in the use of qualitative methods in the field of health economics. This study applies and further develops existing methods in the context of generating economic evidence for precision medicine. It shows a new application of qualitative methods to identify potential capacity constraints for inclusion in the economic evaluation of examples of precision medicine. In addition, this study shows how to apply methods to enable decision analysts to produce evidence to inform if, and how, to implement precision medicine into clinical practice.

This study also demonstrated the application of implementation methods that can be used to adapt existing decision-analytic model–based CEA for examples of precision medicine. The inclusion of 3 capacity constraints, which were identified in qualitative interviews with stakeholders in the implementation process, had the effect of reducing the cost-effectiveness of *ALK* testing and crizotinib and limiting the amount of net societal benefit its introduction produced for the health system.

The results of the analysis of the impact of each individual capacity constraint suggested that constraints could be grouped into 2 main categories based on their impact on the cost-effectiveness or net societal benefit of the intervention. In this study, these categories of constraints will be referred to as *access limiting* or *quality limiting*. The commissioning awareness constraint was an example of an access-limiting constraint. This constraint limited the number of patients who could receive testing, and while this significantly limited the net societal benefit of the intervention, it did not reduce its cost-effectiveness. An access-limiting constraint can therefore be defined as a constraint that reduces the number of patients who receive a potentially cost-effective intervention resulting in a definite change in NMB with no effect on the ICER. The presence of access-limiting capacity constraints that do not affect the ICER highlights the importance of going beyond the use of ICERs and averaged net benefit calculations as the sole outcome of economic evaluation when assessing the impact of capacity constraints. The total population-level NMB calculations used in value of implementation analysis are required to capture the impact of these constraints.

The other type of capacity constraints are quality-limiting capacity constraints. The localization versus centralization and pathology laboratory capacity constraints in this case study were examples of this type of constraint. The impact of these constraints was to either raise the estimated incremental cost or lower the estimated incremental benefit per patient receiving the intervention. Contrary to access-limiting constraints, the presence of these constraints directly affected the cost-effectiveness of *ALK* testing and crizotinib as measured by the ICER. Furthermore, as the incremental costs and QALYs are key to the determination of net benefit and net benefit is a key component of societal net benefit, quality-limiting constraints also reduce the total NMB of the intervention. This can be seen in the estimated individual impacts of the localization versus centralization and pathology laboratory capacity constraints in this case study. In this case study, the impact of the quality-limiting capacity constraints on the estimated ICERs was relatively small. However, this still translated to a loss of QALYs^17^ in the case of limited pathology laboratory capacity.

When combined, the 3 capacity constraints caused a loss of NMB of £5,289,414, which equates to approximately 106 QALYs at the NICE end-of-life threshold. Implementing *ALK* testing and crizotinib in 2014 without addressing any of the constraints would have only yielded 17% of the potential net societal benefit that the test-treat intervention could deliver. While in this case, *ALK* testing and crizotinib were still cost-effective and therefore yielded positive net societal benefits even in the presence of constraints, it is conceivable that quality-limiting capacity constraints could push the ICER for the test-treat intervention above the threshold, resulting in a net societal loss.

Nine published studies incorporating capacity constraints in economic evaluations were identified in a previous systematic review.^4^ There were some similarities with regard to the impact of capacity constraints between the findings of this study and the findings of the 9 studies identified in the systematic review. The study by Retèl et al.^44^ investigating the impact of barriers and facilitators to introducing a Mammaprint test for breast cancer provides a good example of how access-limiting constraints may interact with varying marginal costs and benefits. In this example, Mammaprint has high initial marginal incremental costs and low marginal incremental benefits. The access-limiting constraints of uptake by clinicians, noncompliance with results, and the failure rate of the test acted to limit the number of patients who received Mammaprint, meaning that the ICER for the technology remained high. Over time, the impact of these constraints reduced, increasing the ICER and eventually making the Mammaprint test cost-effective.

Examples of quality-limiting constraints were present in 3 studies^45–47^ identified in the systematic review.^4^ In a study of the impact of adherence to tamoxifen by patients with breast cancer, McCowan et al.^45^ found that patients with an adherence of less than 80% were expected to lose 1.12 QALYs and experience an additional £5970 in medical costs compared with patients with greater than 80% adherence. In 2 studies, the inclusion of quality-limiting constraints had a significant impact on the decision as to which treatment to offer.^46,47^ In a study on NSCLC, when the turnaround time for a multiplexed biomarker test was increased 1.5-fold, the optimal strategy changed from test and then treat to beginning standard chemotherapy before test results were returned.^46^ In an economic evaluation of the cost-effectiveness of letrozole versus tamoxifen for women with breast cancer, letrozole was a dominant option when drug wastage of tamoxifen was 15% but had a positive incremental cost when drug wastage was 0%.^47^

While the test-treat intervention in this case study still created positive net benefits for society, it is possible that for other interventions, the inclusion of capacity constraints will result in the intervention being deemed not cost-effective at the time of evaluation. This may have significant implications for the evaluation of examples of precision medicines by TA agencies such as NICE. Currently, such constraints are not included in economic evaluations, and so the results of such studies should arguably be taken as long-run estimates of cost-effectiveness.^48–50^

There were some limitations to this study that mainly centered around the lack of available data. A significant issue was that there was limited information available about the number of *ALK* tests offered in 2014, the impact of delaying treatment initiation due to a long turnaround time for tests, and the impact of limited pathology laboratory capacity. As such, a number of assumptions had to be made about these values. In addition, it was difficult to determine the mechanism by which the capacity constraints affected the costs, QALYs, and level of provision of *ALK* testing and crizotinib. In future applied studies, robust qualitative methods should be used to explore the mechanisms by which constraints affect cost-effectiveness and the resulting parameter values. This could involve the use of focus groups, the Delphi method, or expert elicitation.^33,51^

This study selected 3 capacity constraints based on the results of a qualitative study. The constraints were selected for the case study model because they were commonly mentioned and had a clear mechanism for how they may affect cost-effectiveness and NMB. However, this does not necessarily mean that these constraints were the ones that had the greatest impact on these outcomes in practice. This may be a particular problem for future prospective studies that seek to incorporate capacity constraints. If many participants anticipate a particular barrier, then they may be prepared to take measures to overcome it when faced with it in practice. More damaging may be the unforeseen constraints that may take longer to overcome.

## Conclusion

This study has demonstrated how value of implementation methods can be used to quantify the impact of health system capacity constraints in an economic evaluation. In this case study, the inclusion of health system capacity constraints significantly reduced the estimated cost-effectiveness and societal net benefit of *ALK* testing and crizotinib. This means that economic evaluations that omit capacity constraints may give misleading representations of the cost-effectiveness of examples of precision medicine. In addition, including measures of total net benefit in economic evaluations that account for capacity constraints may help to highlight situations in which the intervention will not be available to all patients at the time of approval, whereas the ICER does not contain this information.

## Supplemental Material

sj-docx-1-mdm-10.1177_0272989X211053792 – Supplemental material for Quantifying the Impact of Capacity Constraints in Economic Evaluations: An Application in Precision Medicine Supplemental material, sj-docx-1-mdm-10.1177_0272989X211053792 for Quantifying the Impact of Capacity Constraints in Economic Evaluations: An Application in Precision Medicine by Stuart J. Wright, William G. Newman and Katherine Payne in Medical Decision Making
