# Supplementary material for: Quantifying the Impact of Capacity Constraints in Economic Evaluations: An Application in Precision Medicine
Source: Med Decis Making. 2021 Oct 25;42(4):538–53. doi: 10.1177/0272989X211053792 (PMC9005833; doi:10.1177/0272989X211053792)
Supplement: sj-docx-1-mdm-10.1177_0272989X211053792 – Supplemental material for Quantifying the Impact of Capacity Constraints in Economic Evaluations: An Application in Precision Medicine [file sj-docx-1-mdm-10.1177_0272989X211053792.docx]

**Quantifying the Impact of Capacity Constraints in Economic Evaluations: An Application in Precision Medicine**

**Supplementary Appendix A**

This supplementary appendix describes the process of the replication of a published decision-analytic model to estimate the incremental costs, quality adjusted life years and cost-effectiveness of crizotinib compared to docetaxel for the treatment of anaplastic lymphoma kinase positive non-small cell lung cancer. This published decision-analytic model was submitted by a manufacturer to NICE as part of a technology appraisal (TA296). This decision-analytic model provided the baseline results to serve as a comparator for subsequent results produced by a model adapted to incorporate capacity constraints. Table SA.1 outlines the key design criteria of the baseline model.

**Table SA.1:** Key design criteria

| **Decision problems** | What is the cost-effectiveness of *ALK* testing using immunohistochemistry and fluorescent in situ hybridisation to target treatments with crizotinib or docetaxel? |
| --- | --- |
| **Population** | Patients with stage III or IV, EGFR negative non-small cell lung cancer |
| **Intervention** | Anaplastic lymphoma kinase (*ALK)* testing that uses immunohistochemistry (IHC) and fluorescence in situ hybridisation (FISH) to target treatment with an appropriate treatment such as an *ALK* inhibitor (crizotinib). |
| **Comparator** | No testing and a chemotherapy agent (docetaxel). |
| **Model type** | Linked decision tree (testing component) and Markov model (treatment component) |
| **Setting and perspective** | Hospital setting; NHS England |
| **Time Horizon** | Lifetime for this population; 15-years |
| **Costs** | National currency (£) at 2014 prices |
| **Consequences** | Quality adjusted life years (QALYs) |
| **Discounting** | 3.5% for both costs and consequences |
| **Decision rule** | Incremental cost per QALY should fall under £50,000 to be deemed a cost-effective use of resources.  This decision-rule is consistent with end-of-life criteria invoked by NICE for this decision problem as the intervention meets:  List three criteria:   - The treatment is for patients who are expected to live for less than 24 months - The treatment is expected to offer at least 3 additional months of life - The treatment is licenced for a small patient population |

**Baseline model parameters**

Table SA.2 and Table SA.3 provides details of the parameter values used in the baseline analysis of for the decision tree and Markov models.

**Table SA.2:** Parameter values for the decision tree

| **Parameter** | **Value:**  **Base Case** | **Value:**  **PSA**  **Distribution** | **Assumptions** | **Source** |
| --- | --- | --- | --- | --- |
| **Defining the population** | | | | |
| Number of stage III/IV NSCLC patients excluding those with *EGFR* mutations | 16,627 | Fixed | Used population of NSCLC patients from above study and removed 15% who are assumed to test positive for *EGFR* mutations and would therefore receive an *EGFR* TKI | (1) |
| *ALK* mutation prevalence | 3.4% | ~Beta(96,2864) | Originally from Bang et al., (2011), a systematic review of 13 original papers. Value for screening all NSCLC patients rather than just adenocarcinoma | (1,2) |
| **Probabilities** | | | | |
| IHC Sensitivity | 95% | ~U(90,100) | Weighted average of sensitivity across four testing platforms based on values identified in a systematic review. Used value of 95% in analysis as clinicians believed this value would be reached at optimum utilisation | (3) |
| IHC Specificity | 100% | Fixed | Weighted average of specificity across four testing platforms based on values identified in a systematic review. Used value of 100% in analysis as clinicians believed this value would be reached at optimum utilisation | (3) |
| **Unit costs** | | | | |
| IHC testing | £25 | ~U(£23, £28) |  | (1) |
| FISH testing | £120 | ~U(£102,124) | The cost of testing is redacted in Duarte et al., (2013). A value of £120 was suggested in the qualitative interviews in chapter 5. A recent NICE Medtech innovation briefing suggests that the cost is between £100 and £150 | Chapter 5, (4) |

**Table SA.3:** Parameter values for the Markov

| **Parameter** | **Base Case Value** | **Distribution** | **Assumptions** | **Source** |
| --- | --- | --- | --- | --- |
| **Probabilities** | | | | |
| Crizotinib Progression Free Survival | Weibull survival curve  Shape=1.368  Scale=10.554 | Asymptotic normal distribution of survival curve parameter estimates | Based on curve fitted to reproduced KM curve | (5) |
| Crizotinib Overall Survival | Weibull survival curve  Shape=0.952  Scale=34.931 | Asymptotic normal distribution of survival curve parameter estimates | Based on curve fitted to reproduced KM curve | (5) |
| Docetaxel Progression Free Survival | Log-normal survival curve  Mean=1.2735  Standard Deviation=1.0091 | Asymptotic normal distribution of survival curve parameter estimates | Based on curve fitted to reproduced KM curve | (5) |
| Docetaxel Overall Survival | Hazard ratio of 0.627 applied to survival curve for crizotinib OS | ~N(0.33,1.24) | Actual value redacted in publication. Ad hoc value derived from ratio of median OS for docetaxel to median OS for crizotinib | (1) |
| **Utilities** | | | | |
| Crizotinib stable disease utility | 0.82 | 1 minus a disutility drawn from the following distribution  ~beta(114,520) | Based on “on treatment” utility from original trial | (6) |
| Crizotinib progressive disease utility | 0.75 | 1 minus a disutility drawn from the following distribution  ~beta(201,601) | Based on midpoint of range from manufacturers submitted sensitivity analysis | (1) |
| Chemotherapy stable disease utility | 0.74 | 1 minus a disutility drawn from the following distribution  ~beta(216,584) | Based on “on treatment” utility from original trial | (6) |
| Chemotherapy progressive disease utility | 0.57 | 1 minus a disutility drawn from the following distribution  ~beta(449,595) | Based on midpoint of range from manufacturers submitted sensitivity analysis | (1) |
| **Costs** | | | | |
| Monthly cost of Crizotinib | £4,689.00 | Fixed | Original value from 2012 British National Formulary. | (1) |
| Monthly cost of docetaxel | £1,527.86 | U~(£1,375, £1,681) | Original value from 2012 British National Formulary. | (1) |
| Docetaxel administration | £148.20 | ~U(£131, £160) | Figure based on costs included in NICE STA for pemetrexed  Here it is assumed that there is a constant cost per month rather than different costs in first and subsequent months. In original analysis there was only a difference of £0.81 | (1) |
| Treatment of adverse neutropaenia events in chemotherapy arm | £38.16 | ~U(£28.62, £47.70) | Based on 4 (range 3-5) days of treatment with granulocyte colony-stimulating factor at £52.71 per day and an incidence of 18.1% in patients receiving chemotherapy | (1) |
| Routine medical management cost in stable disease state | £241.44 | ~U(£217, £266) | Based on values used in previous NICE TA’s | (1) |
| Routine medical management cost in progressive disease state | £178.09 | ~U(£160, £196) | Based on values used in previous NICE TA’s | (1) |
| Palliative care before death (one off cost in dead state) | £3,923 | Fixed | Based on values used in previous NICE TA’s | (1) |

**Probabilities**

Values for probabilities were required for decision tree and state transition Markov model. The probabilities required for the decision tree reflect the likelihood that a patient receiving *ALK* testing receives a positive or negative result. This was determined by the prevalence of *ALK* mutation in lung cancer patients and the sensitivity and specificity of the IHC and FISH tests. Therefore, IHC and FISH testing parameters were extracted from Djalalov et al (2014) who used values based on a systematic review of 7 studies (3). The authors found a sensitivity of 93% and a specificity of 99%. The authors expected the sensitivity of the test to be better in practice so used a value of 95%. In this study, the 95% value was used in the base case analysis. FISH testing was assumed to have a sensitivity and specificity of 100% as it was the current gold standard test at the time of submission.

**Transition Probabilities**

The probabilities required for the state transition Markov model reflect the likelihood that a patient would transition between health states in a given cycle. These transition probabilities were derived from the durations of PFS and OS for the intervention and comparator. Parametric survival curves were used to populate the transition probabilities in the treatment Markov models. The PFS curves were used to calculate the probability of moving from the progression-free state to the progressive disease state in each one month interval. The OS curve for crizotinib was used to determine the probability of patients moving from either the progression free or progressive disease states to the dead state in a one month time interval. A hazard ratio was applied to this curve to determine the probability of a patient receiving docetaxel moving from either the progression free or progressive disease states to the dead state. The following section outlines the clinical evidence used to inform the transition probabilities in the Markov model.

**Clinical Effectiveness**

Populating the state transition Markov model in terms of clinical effectiveness data was a challenge because of redacted information in the technology appraisal report and the absence of reported scale and shape parameters for the parametric survival curves used to extrapolate progression free survival and overall survival. To address this problem, an approach proposed by Guyot et al. (2012) was used to reconstruct the survival data for crizotinib and docetaxel from published Kaplan-Meier (KM) curves (7). In this approach, the software package DigitizeIt (8) is first used to digitally read a sample of points on a published KM curve to produce a set of underlying data. Data are extracted across various intervals and an additional file is created with details as to these time intervals, the row numbers of the data in the extracted data pertaining to these intervals and the numbers at risk in each period. The algorithm outlined by Guyot et al. (2012) was then applied to these files to incorporate censoring of the data that was assumed to have occurred at a constant rate. The algorithm was applied in the software R (9) by adapting the authors’ published code and involved the use of the “survival” and “flexsurv” packages (10,11). The output of the algorithm is the approximated individual patient data that would have produced the published KM curve.

As KM curves were not published in the ERG group report for crizotinib, they were taken from the published results of the RCTs used in the submission (PROFILE 1005 AND 1007). Progression free survival data for crizotinib and docetaxel were based on the published results of PROFILE 1007 (12). The overall survival data used in the source model were taken from, the then incomplete, PROFILE 1005 study. As these data were not available, this case study used the mature, final overall survival data from PROFILE 1005 (13).

Using the estimated individual-level patient data from the RCTs, parametric survival curves were fitted to extrapolate the data to longer time-frames. These curves model the probability of PFS or OS at a given time period based on different survival distributions such as the exponential, Weibull, and Gompertz distributions among others (14). The same underlying distribution as assumed by the manufacturer for each curve was used in this case study. The same ranking of appropriate assumed distribution, based on model fit statistics, as reported in the source economic evaluation was obtained in this case study. The fitted parametric survival curves for crizotinib PFS (see Figure SA.1), docetaxel PFS (see Figure SA.2) and crizotininb OS (see Fgure SA.3) were generated using the “ggplot2” package in R (15).

**Figure SA.1:** Weibull survival curve for progression free survival with crizotininb
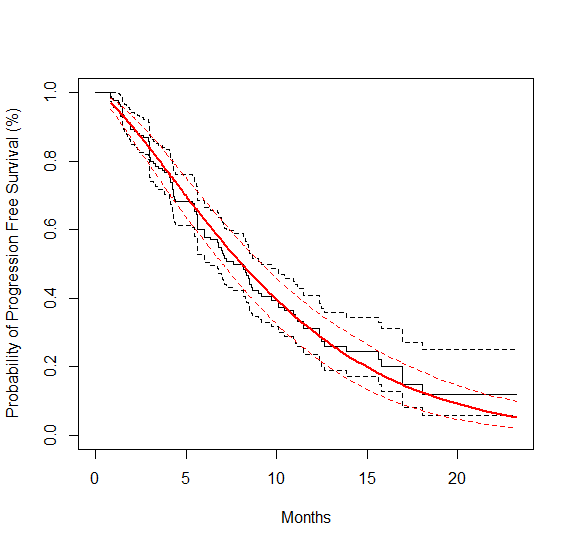


**Footnote**: the black solid line indicates the Kaplan-Meier curve created from the reconstructed survival data while the black dotted lines represent the confidence intervals around this curve. The red solid line represents the survival curve estimated from the specified parametric survival curve while the red dotted lines represent the confidence intervals around this curve.

**Figure SA.2: Log-normal survival curve for progression free survival with docetaxel**


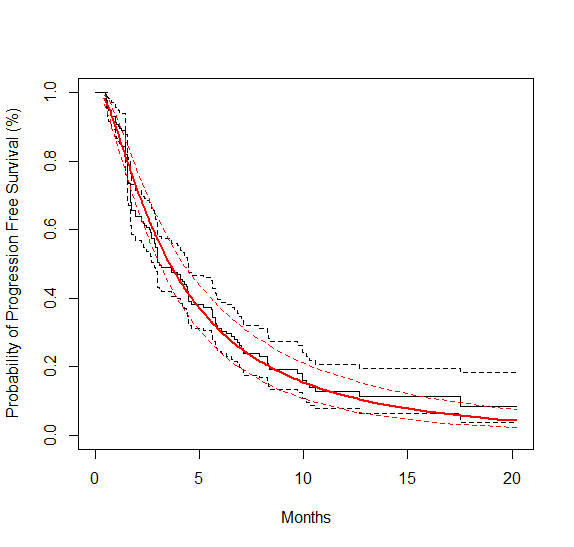


**Footnote:** the black solid line indicates the Kaplan-Meier curve created from the reconstructed survival data while the black dotted lines represent the confidence intervals around this curve. The red solid line represents the survival curve estimated from the specified parametric survival curve while the red dotted lines represent the confidence intervals around this curve.

**Figure SA.3:** Weibull survival curve for overall survival with crizotinib


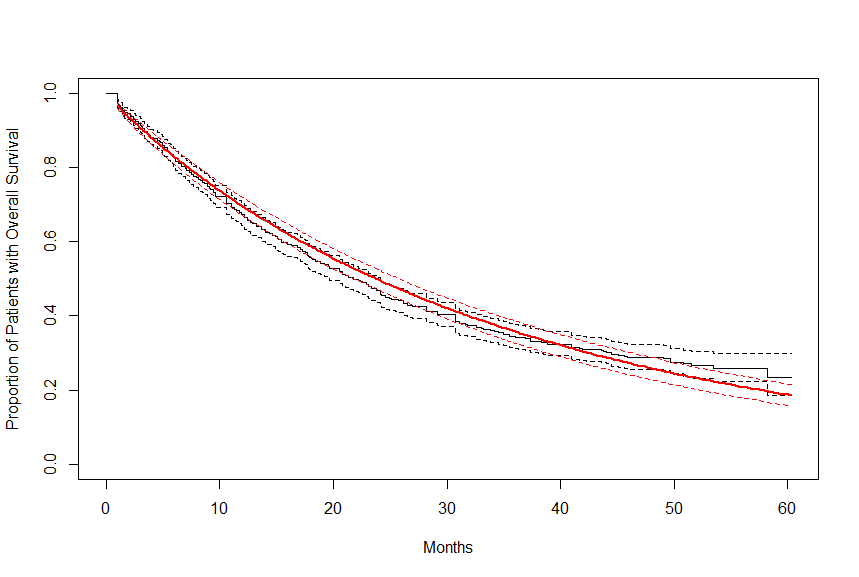


Footnote: the black solid line indicates the Kaplan-Meier curve created from the reconstructed survival data while the black dotted lines represent the confidence intervals around this curve. The red solid line represents the survival curve estimated from the specified parametric survival curve while the red dotted lines represent the confidence intervals around this curve.

To calculate the OS of patients receiving docetaxel, Pfizer applied a hazard ratio (HR) to the parametric survival curve for crizotinib. The hazard ratio measures the rate of survival in one group relative to another at any time point (14). However, the value of this HR was redacted in the published Evidence Review Group report (1). In this case study the HR was approximated by taking the ratio of reported median overall survival in the docetaxel group (20.7 months) to that of those in the crizotinib group (33 months). This resulted in a value of a HR of 0.627.

**Costs**

All prices are reported in 2014 pounds sterling. There were two main types of relevant NHS costs (for the test pathway and for the medicine pathway). A cost of IHC testing of £25 was included in the published NICE TA296 but the cost of FISH testing was redacted. In this case study, a value of £120 was used based on a value stated in a subsequent NICE Technology Appraisal (4).

Cost data for both the intervention and comparator treatments were readily available in the published NICE TA296. The monthly cost of crizotinib was £4,689 while docetaxel was £1,528. As docetaxel is delivered intravenously in hospital it had an administration cost of £148.20 per month. Adverse events were similar across intervention and comparator with the exception of higher rates of neutropaenia in those patients receiving chemotherapy. A cost of £38.16 was included for treating this neutropaenia based on a calculation included in the Evidence Review Group report of four (range three to five) days of treatment with granulocyte colony-stimulating factor at £52.71 per day and an incidence of 18.1% in patients receiving chemotherapy. Routine medical management costs in the stable and progressive states of both the intervention and comparator were £241.44 and £178.09 respectively. A one-time palliative care cost of £3,923 was applied at time of death for each patient regardless of their treatment modality. This value was identified by calculating the additional number of weeks spent in the dead state in each cycle in each arm to represent the number of patients who had died. This figure was multiplied by the discounted cost of palliative care and added to the total expected cost for the intervention and comparator.

**Consequences**

The relevant consequences used in this case study, consistent with the NICE reference case, was the quality-adjusted life years (QALYs) gained by patients receiving the intervention or comparator. QALYs comprise two components: health-related quality of life (HRQoL), and length of life. The latter was determined by the duration in which patients were in either the progression free or progressed disease health states as opposed to the death health state.

HRQoL is measured using a health state utility value between 1 for full health and 0 for death. Negative values are also possible for health states which are worse than death. The impact on health was assumed to be a consequence of the medicine not the testing process. Utility values for cancer health states in the treatment Markov models were redacted in the published Evidence Review Group critique. Utility values were instead taken from published results of the PROFILE 1007 study used in the source model (6). On-treatment utility values were used to represent the utility value of the progression free state (crizotinib=0.82, docetaxel=0.74). The utility values used for the progressed disease state in the source model were taken from the average utility of patients on ending treatment in PROFILE 1007. However, these values were not published in the journal article so values for this state were calculated by taking the midpoint value of the lower and upper bound utility values presented in deterministic sensitivity analysis in the source model (1). This corresponded to a value of 0.75 for crizotinib and 0.57 for docetaxel.

**Analysis**

The analysis was divided into three parts: the base case analysis; sensitivity analysis; and a comparison of the outcomes of the model with those of the model it sought to replicate.

**Base Case Analysis**

In the base case analysis for this study point estimate values for each model input parameter were used to determine the incremental costs and consequences of testing NSCLC patients for NSCLC mutations and then using targeted treatment compared with treating all patients with docetaxel.

*Determining the Incremental Cost-Effectiveness of the Intervention*

An estimate of the cost-effectiveness of these interventions was provided by calculating the incremental cost-effectiveness ratio (ICER) for the intervention (see equation 1). This estimate represents the cost per additional quality-adjusted life year gained from the intervention relative to the comparator and is calculated using the formula below:

$$\begin{aligned} \boldsymbol{ICER=}\frac{\left( \boldsymbol{Cost}_{\boldsymbol{2}}\boldsymbol{-}\boldsymbol{Cost}_{\boldsymbol{1}} \right)}{\left( \boldsymbol{QALY}_{\boldsymbol{2}}\boldsymbol{-}\boldsymbol{QALY}_{\boldsymbol{1}} \right)} \#\left( Equation 1 \right) \end{aligned}$$

Where $C_{2}$ is the expected total cost of the intervention, $C_{1}$ is the expected total cost of the comparator, ${QALY}_{2}$are the expected total QALYs experienced by those receiving the intervention, and ${QALY}_{1}$ are the expected total QALYs experienced by those receiving the comparator.

In the technology appraisal for crizotinib, the NICE end-of-life threshold of £50,000 was used as the benchmark for cost-effectiveness. This threshold is used for interventions where there is small patient population, with patients who are expected to have less than 2 years left to live, and whose life expectancy is likely to be extended by at least 3 months with use of the intervention (16).

*Determining Total Net Monetary Benefit of the Intervention*

The net monetary benefit of treating all patients with the intervention was calculated to generate an estimate of the total yearly value of the intervention to the NHS produced by the intervention. The yearly value of the intervention is the expected net monetary benefit per patient multiplied by the number of patients. The estimate produced will be the value of perfect implementation as economic evaluations currently implicitly assume all patients immediately receive the intervention following approval. Equation 2 outlines how the net monetary benefit of perfect implementation of the interventions was calculated:

$$\begin{aligned} \mathbf{Value of Perfect Implementation}= \\ \boldsymbol{n}\left( \boldsymbol{k.\Delta}\boldsymbol{QALY}_{\boldsymbol{p=1}}\boldsymbol{-\Delta}\boldsymbol{C}_{\boldsymbol{p=1}} \right)\boldsymbol{-n.p}\left( \boldsymbol{k.\Delta}\boldsymbol{QALY}_{\boldsymbol{p}}\boldsymbol{-\Delta}\boldsymbol{C}_{\boldsymbol{p}} \right) \#\left( \mathrm{Equation}2 \right) \end{aligned}$$

$(Where k=£50,000)$

Alternatively this can be replace more simply by substituting in NMB which is fixed in the base case analysis:

$$\begin{aligned} \mathbf{Value of Perfect Implementaiton}\boldsymbol{= n}\left( \boldsymbol{NMB}_{\boldsymbol{p=1}} \right)\boldsymbol{-n.p}\left( \boldsymbol{NMB}_{\boldsymbol{p}} \right)\#\left( \mathrm{Equation} 3 \right) \end{aligned}$$

or

$$\mathbf{Value of Perfect Implementaiton}\boldsymbol{=n}\boldsymbol{(NMB}_{\boldsymbol{p=1}}\boldsymbol{-}\boldsymbol{p.NMB}_{\boldsymbol{p}}\boldsymbol{)}$$

As in this example it is assumed that implementation goes from zero to 100% upon approval then this reduces to:

$$\begin{aligned} \mathbf{Value of Perfect Implementaiton}= \boldsymbol{n}\left( \boldsymbol{NMB}_{\boldsymbol{p=1}} \right)\#\left( \mathrm{Equation} 4 \right) \end{aligned}$$

*Sensitivity analysis*

To account for parameter uncertainty affecting the estimated ICERs and value of the interventions, probabilistic sensitivity analysis was undertaken. The distributions used for each parameter are outlined for the decision tree (Table 2) and state transition Markov model (Table 3). In this case study, 1,000 such Monte Carlo simulations were completed. The ICERs estimated in each iteration were used to generate net monetary benefit values. The proportion of simulations with positive net benefit estimates in each iteration was then used to calculate the probability that the intervention would be cost-effective.

Evidence on the assumed distribution of the values of key parameters was rarely available in the published Evidence Review Group report on the submission by Pfizer. The distribution of prevalence rate of *ALK* mutations was reported based on a published review (2). The sensitivity of IHC testing reported in Djalalov et al., (2014) featured upper and lower bounds but no information about the distribution so a uniform distribution was applied to these bounds of the parameter estimate. The recreation of the survival curves from the published KM curves meant that estimates of the survival distributions could be created. As the hazard ratio for overall survival for docetaxel was produced using an approximation, a true distribution could not be estimated. Instead, this analysis used the reported lower and upper bounds for deterministic sensitivity analysis conducted by the manufacturer to represent confidence intervals for this variable. From these, a normal distribution of hazard ratios was created as an approximation for the distribution of hazard ratios. This approach was also used for the cost parameters, although uniform distributions were used to provide conservative over-estimates of the potential uncertainty. For the utility estimates for the progression free disease states, confidence intervals were available from the published results of the PROFILE 1007. Beta distributions were fitted using the mean utility and these confidence intervals. For the approximated utility values used for the progressive disease state, it was assumed that the confidence intervals would be of the same magnitude and these were calculated and used to create additional beta distributions for these parameters.

*Model Validation*

The evidence provided by decision-analytic model based economic evaluations informs decisions as to the allocation of significant levels of health system resources with resulting changes in the health of different groups of patients. As such it is important to evaluate the validity of decision-analytic models. To determine the validity of the case study model, the Assessment of the Validation Status of Health-Economic decision models (ADVISHE) model validation checklist was applied (17). This checklist poses a series of questions to modellers about various aspects of model validity including in the conceptual model, parameters used and the cross validity of the model and results. However, as the aim of this model was to replicate the manufacturer submission by Pfizer for the NICE Technology Appraisal of crizotinib (NICE TA 296) (1), some aspects of validity will not be relevant in this case study while others will be of greater importance. For example, it is assumed that the manufacturers would have addressed some components of model validation: validation of the conceptual model (Part A of the AdViSHE checklist) and input data validation in the source model (Part B of the AdViSHE checklist). In this case study, model validation therefore focused: validation of the computerised (case study) model (Part C of the AdViSHE checklist) and operational validation (Part D of the AdViSHE checklist). The key purpose of the model validation conducted in this study was to ensure the results of the baseline model were similar to the results produced in the source model. The outputs on which the validity of the model were judged were the expected costs and QALYs of the intervention and comparator per patient treated, the incremental costs and benefits, and the ICERs.

**Results**

This section presents the base case results; results of the sensitivity analysis; and a comparison of the outcomes of the model with those of the model it sought to replicate.

*Base Case Analysis*

In the base case analysis, the use of *ALK* testing to guide treatment with crizotinib or docetaxel had an incremental cost of £1,391 (using a price year of 2014) and provided an additional 0.035 QALYs per patient tested. When framed in terms of only *ALK* positive patients, as in the source model, crizotinib provided an additional 1.044 QALYs at an incremental cost of £40,161. Depending on this framing, the ICER for the intervention was £39,198 or £38,468 respectively. The higher ICER when accounting for the testing process results from the cost of testing and imperfect nature of the test. For example, 16,032 patients received negative results from the original IHC test whilst 30 received initial positive results which were later determined to be false-positive by FISH tests. Such patients incur a cost of testing but receive no expected benefit so they serve to inflate the ICER.

This case study used the example of *ALK* testing to inform prescribing of crizotinib and the ICER of £39,198 per QALY represents the cost-effectiveness of the intervention. Given this ICER, there is evidence to suggest that *ALK* testing and treatment with crizotininb or docetaxel is cost-effective assuming the NICE end-of-life threshold of £50,000 per QALY. At this level of cost-effectiveness, the intervention would provide a value (total NMB) of £6,373,887 per year. Figure SA.4 shows the value of the intervention at different cost-effectiveness thresholds.

**Figure SA.4:** Total incremental monetary net benefit of *ALK* testing to guide treatment with crizotinib or docetaxel by threshold level


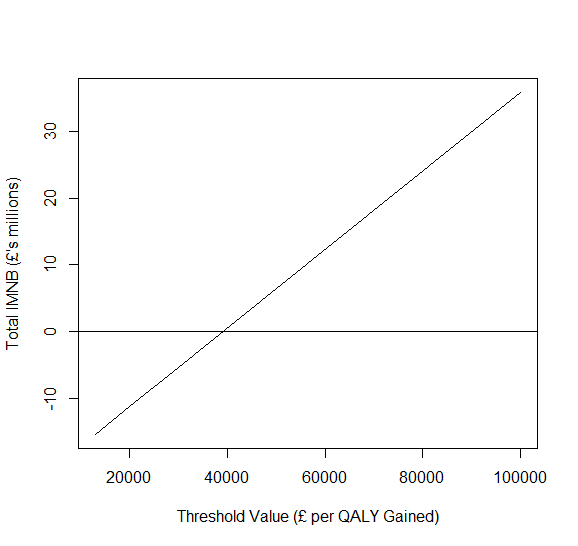


*Sensitivity Analysis*

The probabilistic sensitivity analysis for this case study suggested that there was an 85% probability that *ALK* mutation testing and targeted treatment with crizotinib was cost-effective assuming the NICE end-of-life threshold. Figure SA.5 shows the cost-effectiveness acceptability plane with the 1,000 simulated incremental costs and benefits. Figure SA.6 shows the distribution of total annual value estimates for the intervention.

**Figure SA.5:** Distribution of Estimated Incremental Costs and Benefits


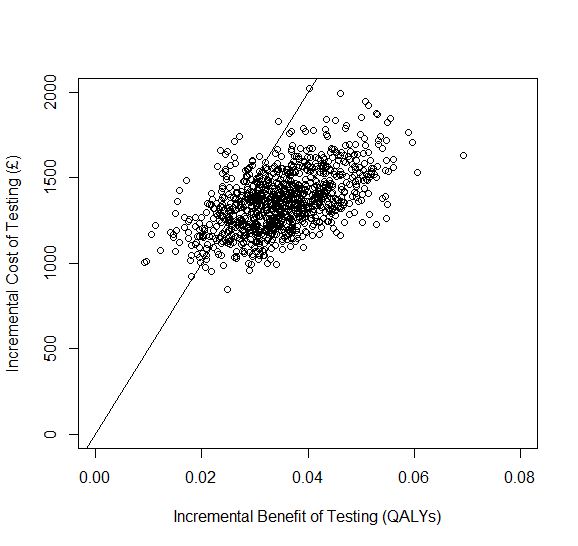


**Figure SA.6:** Distribution of Annual Total Expected Net Monetary Benefit


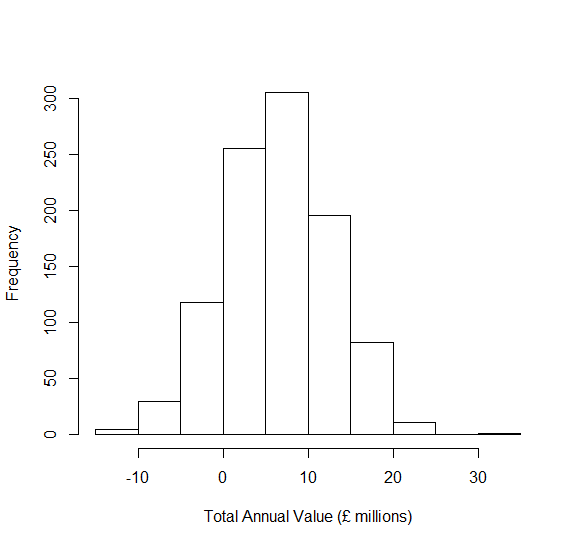


*Model Validity*

To determine the validity of the case study model, Part C and D of the ADVISHE checklist were used to critique the model and the estimated results (17). Part C of the checklist concerns validation of the technical production of computerised model to check for coding errors. This was completed by a second researcher.

To further check for coding errors, the model was run with various extreme parameter values including large ranges in drug cost, utility values for stable and progressive disease states, and differing parametric survival curves. The component decision tree and state transition Markov model were individually checked for errors. The conditional probabilities for the decision tree were calculated and manually checked. These conditional probabilities were applied to the cohort to determine patient flows into the different Markov models and these numbers exhibited validity when compared to the estimated number of patients receiving crizotinib in the source model. The Markov traces for each treatment option were examined and plotted over time for evidence of coding errors.

Part D of the ADVISHE checklist focuses on operational validity. The key area of validity for the case study model is in cross validation testing of model outcomes when compared with the source model. For the case study model to be valid, it should estimate similar estimates of deterministic expected cost and consequences to those produced by the source models for patients receiving either the intervention or comparator. As the decision problem used in the case study differed slightly to that in the source model, the expected cost and QALYs for patients who had received testing and were *ALK* positive and received crizotininb were compared with those patients who had tested negative and received docetaxel (see Table SA.4).

**Table SA.4:** Comparison of the deterministic results from this case study with published manufacturer estimates

| *ALK* Testing and Targeted Therapy with Crizotinib | | |
| --- | --- | --- |
| Parameter | Source model^a^ | Case Study Model (Percentage difference) |
| Mean Total Cost for Patients Receiving Docetaxel | £13,922 | £14,748 (+6%) |
| Mean Total Cost for Patients Receiving Crizotinib | £54,149 | £54,908 (+1%) |
| Mean Total QALYs for Patients Receiving Docetaxel | 0.981 | 1.049 (+7%) |
| Mean Total QALYs for Patients Receiving Crizotinib | 1.949 | 2.093 (+7%) |
| Incremental Costs | £40,227 | £40,161 (-0.2%) |
| Incremental QALYs | 0.968 | 1.044 (+8%) |
| ICER (£/QALY) | £41,554 | £38,468 (-7%) |

^a^ (1)

The expected costs and QALYs produced in this case study slightly underestimated the ICER produced in the source model. This effect appears to mainly be driven by larger estimated QALYs and an increase in the difference between these estimates. One explanation for this effect may be that the mid-point values taken from the published confidence intervals which were used to estimate some utility values in the case study model were overestimates of the deterministic health utility. It may be that the distribution of HRQoL for patients with NSCLC is skewed to the left, meaning that patients are more likely to have more severe symptoms, with fewer patients experiencing mild symptoms. This would have meant that the true average health utility for some of the health states may have been lower, reducing the total QALYs experienced in both arms.

The probability of cost-effectiveness, estimated in the probabilistic sensitivity analysis, was greater than the 65% estimated in the manufacture submission by Pfizer but this is expected given the lower ICER estimated in the case study. Additional differences are likely driven by the distribution used for the hazard ratio of overall survival for docetaxel relative to crizotinib. This value was only an approximation in the deterministic analysis and was shown to be the critical variable in the source model. In this analysis the distribution of the ratio was not available so a normal distribution was chosen for pragmatic reasons. However, other distributions will lead to very different probabilities of cost-effectiveness. For example, using a uniform distribution bound by the intervals reported in the deterministic sensitivity analysis in the source economic evaluation yields a probability of cost-effectiveness of only 49%.

**References**

1. Duarte A, Burch J, Smith A, Walker S, Fox D, Rodriguez-Lopez, et al. Crizotinib for ALK fusion positive NSCLC : ERG report Crizotinib for ALK fusion positive NSCLC : ERG report. York; 2013.

2. Bang Y-J. The potential for crizotinib in non-small cell lung cancer: a perspective review. Ther Adv Med Oncol [Internet]. 2011 [cited 2019 Jul 15];3(6):279. Available from: https://www.ncbi.nlm.nih.gov/pmc/articles/PMC3210468/

3. Djalalov S, Beca J, Hoch JS, Krahn M, Tsao M-SS, Cutz J-CC, et al. Cost effectiveness of EML4-ALK fusion testing and first-line crizotinib treatment for patients with advanced ALK-positive non-small-cell lung cancer. J Clin Oncol [Internet]. 2014 Dec 28 [cited 2016 Dec 8];32(10):1012–9. Available from: http://ovidsp.ovid.com/ovidweb.cgi?T=JS&PAGE=reference&D=med8&NEWS=N&AN=24567430

4. National Institute for Health and Care Excellence. HTG EdgeSeq ALKPlus Assay EU for ALK status testing in non-small-cell lung cancer [Internet]. 2017 [cited 2018 Dec 4]. Available from: https://www.nice.org.uk/terms-and-

5. Shaw AT, Kim D-W, Nakagawa K, Seto T, Crinó L, Ahn M-J, et al. Crizotinib versus Chemotherapy in Advanced ALK-Positive Lung Cancer. N Engl J Med [Internet]. 2013 Jun 20 [cited 2019 May 15];368(25):2385–94. Available from: http://www.ncbi.nlm.nih.gov/pubmed/23724913

6. Blackhall F, Kim D-W, Besse B, Nokihara H, Han J-Y, Wilner KD, et al. Patient-Reported Outcomes and Quality of Life in PROFILE 1007: A Randomized Trial of Crizotinib Compared with Chemotherapy in Previously Treated Patients with ALK-Positive Advanced Non–Small-Cell Lung Cancer. J Thorac Oncol [Internet]. 2014 Nov 1 [cited 2019 May 15];9(11):1625–33. Available from: https://www.sciencedirect.com/science/article/pii/S1556086415307292?via%3Dihub

7. Guyot P, Ades A, Ouwens MJ, Welton NJ. Enhanced secondary analysis of survival data: reconstructing the data from published Kaplan-Meier survival curves. BMC Med Res Methodol [Internet]. 2012 Dec 1 [cited 2019 May 15];12(1):9. Available from: https://bmcmedresmethodol.biomedcentral.com/articles/10.1186/1471-2288-12-9

8. Bormann I. DigitizeIt [Internet]. 2019. Available from: https://www.digitizeit.de/contact.html#Contact

9. R Core Team. R: A language and environment for statistical computing [Internet]. Vienna, Austria; 2019. Available from: https://www.r-project.org

10. Thernau T. A Package for Survival Analysis in S_ [Internet]. 2015. Available from: https://cran.r-project.org/package=survival

11. Jackson C. flexsurv: A Platform for Parametric Survival Modeling in R. J Stat Softw. 2016;70(8):1–33.

12. Shaw AT. Effect of treatment duration on incidence of adverse events (AEs) in a phase III study of crizotinib versus chemotherapy in advanced ALK positive non-small cell lung cancer (NSCLC). In: World Conference of Lung Cancer 2013 [Internet]. 2013. Available from: http://library.iaslc.org/search-speaker?search_speaker=20660

13. Blackhall F, Camidge DR, Shaw AT, Soria J-C, Solomon BJ, Mok T, et al. Final results of the large-scale multinational trial PROFILE 1005: efficacy and safety of crizotinib in previously treated patients with advanced/metastatic ALK-positive non-small-cell lung cancer. ESMO Open [Internet]. 2017 [cited 2019 May 15];2:219. Available from: http://esmoopen.bmj.com/

14. Latimer N. NICE DSU TECHNICAL SUPPORT DOCUMENT 14: SURVIVAL ANALYSIS FOR ECONOMIC EVALUATIONS ALONGSIDE CLINICAL TRIALS -EXTRAPOLATION WITH PATIENT-LEVEL DATA REPORT BY THE DECISION SUPPORT UNIT. 2011.

15. Wickham H. ggplot2: Elegant graphics for Data Analysis. 1st ed. New-York: Springer-Verlag; 2009.

16. National institute for Health and Clinical Excellence. Appraising life-extending, end of life treatments [Internet]. 2009 [cited 2017 Jan 20]. Available from: https://www.nice.org.uk/guidance/gid-tag387/documents/appraising-life-extending-end-of-life-treatments-paper2

17. Vemer P, Corro Ramos I, van Voorn GAK, Al MJ, Feenstra TL. AdViSHE: A Validation-Assessment Tool of Health-Economic Models for Decision Makers and Model Users. Pharmacoeconomics. 2016 Apr 1;34(4):349–61.
